# Conditional survival analysis and real-time prognosis prediction for prostate cancer patients

**DOI:** 10.1038/s41598-025-00420-9

**Published:** 2025-05-28

**Authors:** Li Xu, Xudong Mao, Yiming Ding, Zhenwei Zhou, Yuanlei Chen, Mingchao Wang, Gonghui Li, Zeyi Lu

**Affiliations:** Department of Urology, Sir Run Run Shaw Hospital, Zhejiang University School of Medicine, Hangzhou, 310016 China

**Keywords:** Prostate cancer, Conditional survival, Cancer-specific survival, Prognosis, Nomogram, SEER, Prostate cancer, Cancer screening

## Abstract

To assess shifts in long-term survival outcomes for prostate cancer patients, the present study conducted conditional survival (CS) analysis and developed a personalized CS-nomogram to provide dynamic prognostic predictions. Data from 301,441 prostate cancer patients in the Surveillance, epidemiology, and end results (SEER) database were analyzed, with cancer-specific survival (CSS) estimated using the Aalen Johansen estimator. Key predictive factors, including tumor size, lymph node involvement, distant metastasis, and histological grade, were identified via LASSO and multivariable Cox regression analyses to construct the nomogram. Results showed an increase in 12-year CSS from 94.3% at diagnosis to 99.4% for those surviving more than 5 years, with prostate cancer-specific mortality falling below 5% after 5–6 years of survival. The CS-nomogram demonstrated high predictive accuracy, achieving a concordance index (C-index) of 0.869 and area under the curve (AUC) values between 0.847 and 0.904 over a 12-year follow-up period, which provides dynamic, individualized prognostic estimates, supporting the long-term management of prostate cancer patients.

**Supplementary Information:**

The online version contains supplementary material available at 10.1038/s41598-025-00420-9.

## Introduction

Prostate cancer, a major malignant cancer in the urinary system has seen a global rise, accounting for a crucial morbidity and mortality for men^1,2^. Its treatment and prognosis are largely based on the pathologic evaluation of a prostate specimen. Recent studies suggest that prostate-specific antigen (PSA) screening may help to reduce prostate cancer mortality rate^3,4^. Receiving more accurate data on survival assessment has become a matter of increasing concern. However, conventional methods for predicting survival rates based on the pathological stage or Gleason score were not able to provide current survival predictions for patients who could potentially live for many years^5,6^. Currently, studies have shown notable advancements in extended survival rates over the years, with a discrepancy in the 10-year survival rate projected at diagnosis compared to after 8 years of patient survival^7,8^. Thus, traditional survival models could not provide such dynamic and real-time prognostic estimates.

Conditional survival (CS) is a valuable metric for the assessment of survival over time, indicating the likelihood that a patient who has lived for x years following the initial diagnosis of prostate cancer will continue to survive for an additional y year^9,10^. This metric has been applied in gastrointestinal malignancies, including pancreatic adenocarcinoma^11,12^, gastric cancer^13^, liver cancer^14^ and colorectal cancer^15^. The survival prediction tool may assist clinicians in risk stratification and patient counseling by providing individualized prognostic estimates, which can support shared decision-making in clinical practice.

Although traditional nomograms are able to personalize prognosis prediction^16–18^, they are unable to show the evolution of survival over time like CS does. Nevertheless, the CS is flawed since it lacks individualized prognostic factors. Using CS in a nomogram may be promising for personalized and dynamic prognostic prediction. A novel CS-nomogram could potentially address the limitations of both CS and nomograms. This study aimed to evaluate conditional cancer-specific survival (C-CSS) in prostate cancer patients and develop a CS-nomogram to provide precise and up-to-date prognostic data for long-term survivors.

## Materials and methods

The dataset of prostate cancer patients in this research was obtained from the SEER Program’s April 2019 update by the National Cancer Institute^19^. SEER gathers information on cancer occurrence and patient outcomes from 21 areas, covering details like patient characteristics, diagnosis, and survival data, such as age at diagnosis, sex, location of primary tumor, and tumor stage. These data are routinely collected and publicly available in an anonymized format. The predictors included in the study are race, histology, grade, tumor infiltration degree, lymph node involvement, presence of distant metastasis, primary Gleason score, secondary Gleason score, surgery, radiotherapy, age, and PSA. The tumor infiltration degree, lymph node involvement, and presence of distant metastasis are evaluated using the American Joint Committee on Cancer (AJCC) TNM staging system.

**Fig. 1:**
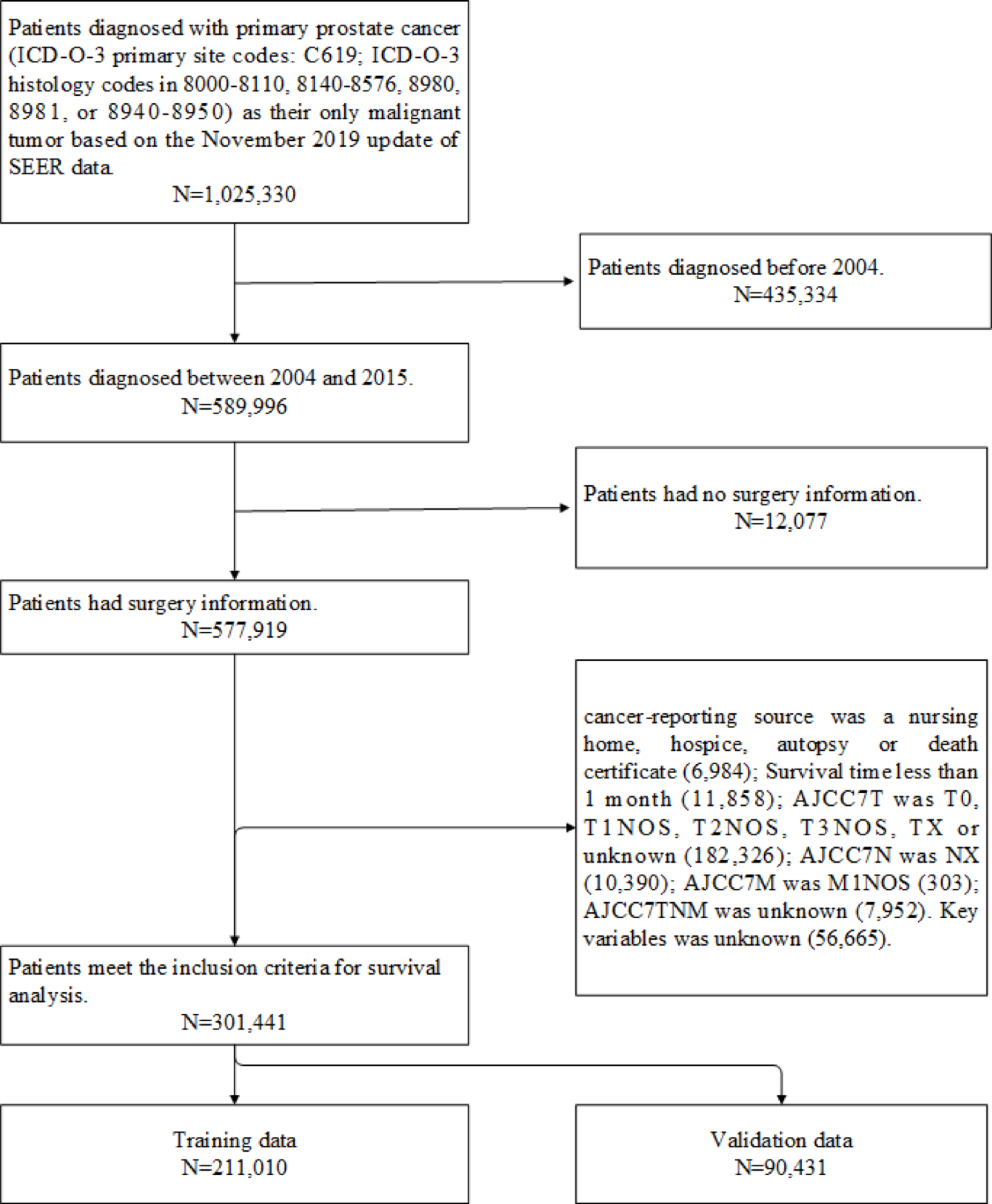
Flow chart for screening patients with prostate cancer. SEER, the surveillance, epidemiology, and end results. AJCC7T means the T stage of the AJCC 7th edition staging; AJCC7N means the N stage of the AJCC 7th edition staging; AJCC7M means the M stage of the AJCC 7th edition staging; AJCC7TNM means the TNM stage of the AJCC 7th edition staging.

The inclusion criteria were: (1) patients diagnosed with primary prostate cancer (SEER primary site code C619, ICD-O-3 histology codes in 8000–8110, 8140–8576, 8980, 8981, or 8940–8950); (2) only one malignant tumor; (3) diagnosed between 2004 and 2015. The time frame of 2004 to 2015 was selected due to the implementation of the Collaborative Stage Data Collection System in 2004, which guaranteed uniform and reliable reporting of cancer staging and coding elements, with 2015 marking the end of the follow-up period. Patients were excluded for: (1) unknown if surgery performed; (2) tumor diagnosis from nursing homes, hospice hospitals, autopsies, or death certificates; (3) survival duration under 1 month; 3) in situ cancer; (4) incomplete data on diagnosis age, gender, tumor infiltration depth, lymph node involvement, distant metastasis, grade, primary and secondary Gleason scores, and PSA levels. In the end, the chosen patient data was split into training and validation sets at a ratio of 7–3 using stratified random sampling based on CSS. A random seed was used to ensure the reproducibility of the sampling process. The specific data selection process is illustrated in the Fig. 1.

The clinical endpoint in this study is cancer-specific mortality, defined as death of the patient due to prostate cancer. C-CSS is determined by dividing the cancer-specific survival at a certain time point by the cancer-specific survival at an earlier time point, which can be expressed as the formula: C-CSS(y|x) = CSS(y + x)/CSS(x). In this formula, C-CSS(y|x) represents the probability of a patient surviving additional y years after already surviving x years following a prostate cancer diagnosis. Additionally, CSS(x) and CSS(y + x) represent the likelihood of surviving cancer at x years and (x + y) years, respectively, calculated using the Aalen Johansen estimator.

In the training set, LASSO regression was used to select clinicopathological factors influencing CSS in patients. The variables selected by LASSO were then included in a multivariable Cox regression analysis. Following the acquisition of the multivariable Cox model, the hazard ratio (HR) and the corresponding confidence interval for each variable were computed. A nomogram was created to visually assess the prognostic risk for patients using the findings from the multivariable Cox regression analysis. Additionally, the conditional survival formula was incorporated into the nomogram, allowing the model to provide personalized dynamic predictions of survival time based on the disease characteristics of prostate cancer patients. The survival nomogram calculates scores based on predictive factors; when a patient’s individual variables are entered, a total score is generated that correlates with the patient’s likelihood of survival. Crucially, the survival data is consistently refreshed with the passage of time.

Continuous variables that adhere to a normal distribution are displayed as mean ± standard deviation, whereas those that do not adhere to a normal distribution are displayed as median (interquartile range). The normality of the data was assessed using Kolmogorov–Smirnov test. For normally distributed data, comparisons between two groups are performed using the independent samples t-test. When dealing with data that is not normally distributed, the Mann–Whitney U test is employed to compare groups. Categorical variables are described using frequency and percentage, with group differences compared using the chi-square test. A two-sided test with *P* < 0.05 is considered statistically significant.

The model’s predictive performance was assessed in both the training and validation datasets through the analysis of ROC curves, calibration curves, and decision curve analysis (DCA). ROC curves were generated over time, and the corresponding AUC values were computed to evaluate the predictive accuracy of the model at various time intervals. Calibration plots were utilized for assessing the precision of the model, where calibration curves approaching the optimal 45° line suggest that the nomogram-predicted probabilities closely align with the real values. The clinical usefulness of the nomogram was evaluated through DCA, which measures the overall advantage of its use. R software version 4.2.3 was utilized for statistical analysis. The specific packages and their functions are as follows: the rms package was used for Cox regression analysis and constructing the nomogram, the risk Regression package was used for plotting ROC and calibration curves, and the d curves package was used for drawing DCA curves.

## Results

### Clinicopathological characteristics of prostate cancer patients

**Fig. 2:**
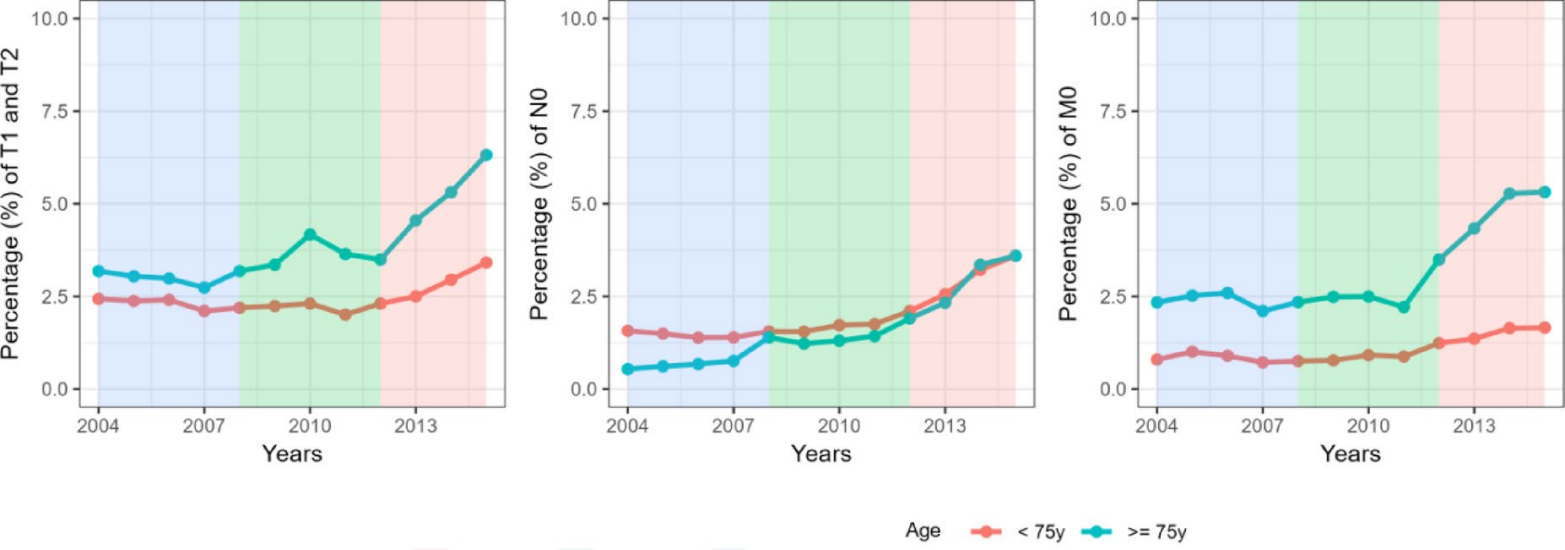
The percentage of T, N, M staging changed over time.

This study involved 301,441 individuals diagnosed with prostate cancer between 2004 and 2015, with 211,010 assigned to the training group and 90,431 to the validation group. The detailed inclusion and exclusion criteria was shown in the Fig. 1. Patients had a median age of 65.0 years (IQR [59.0,70.0]), with a median follow-up of 5.92 years (IQR 3.42–8.83 years) and were mostly white (76.3%). The pathological type was predominantly adenocarcinoma in 98.9%. Upon diagnosis, the majority of patients presented with Gleason score not greater than 6, meanwhile 98.7% had no distant metastases, and a small percentage (1.9%) had lymph nodes metastases. Refer to Table 1 for more information. We analyzed the incidence of TNM staging in the patient population between 2004 and 2015. The results (Fig. 2) showed that the proportion of advanced T, N, M stages changed over time, with a notable increase observed after 2010.

**Table 1:** Clinicopathological characteristics of prostate cancer patients.

	Level	Overall	Training data	Validation data	*p*
N		301,441	211,010	90,431	
Race	White	229,900 (76.3)	160,940 (76.3)	68,960 (76.3)	0.332
	Black	50,187 (16.6)	35,208 (16.7)	14,979 (16.6)	
	Others	21,354 ( 7.1)	14,862 ( 7.0)	6492 ( 7.2)	
Histology	Adenocarcinoma	298,206 (98.9)	208,719 (98.9)	89,487 (99.0)	0.316
	Others	3235 ( 1.1)	2291 ( 1.1)	944 ( 1.0)	
Grade	Grade I	18,190 ( 6.0)	12,727 ( 6.0)	5463 ( 6.0)	0.758
	Grade II	138,828 (46.1)	97,234 (46.1)	41,594 (46.0)	
	Grade III	143,947 (47.8)	100,706 (47.7)	43,241 (47.8)	
	Grade IV	476 ( 0.2)	343 ( 0.2)	133 ( 0.1)	
T	T1	244,090 (81.0)	171,020 (81.0)	73,070 (80.8)	0.448
	T2	49,494 (16.4)	34,524 (16.4)	14,970 (16.6)	
	T3	6248 ( 2.1)	4343 ( 2.1)	1905 ( 2.1)	
	T4	1609 ( 0.5)	1123 ( 0.5)	486 ( 0.5)	
N	N0	295,582 (98.1)	206,875 (98.0)	88,707 (98.1)	0.340
	N1	5859 ( 1.9)	4135 ( 2.0)	1724 ( 1.9)	
M	M0	297,472 (98.7)	208,221 (98.7)	89,251 (98.7)	0.266
	M1a	329 ( 0.1)	238 ( 0.1)	91 ( 0.1)	
	M1b	2958 ( 1.0)	2092 ( 1.0)	866 ( 1.0)	
	M1c	682 ( 0.2)	459 ( 0.2)	223 ( 0.2)	
Gleason score	<= 6	137,484 (45.6)	96,284 (45.6)	41,200 (45.6)	0.690
	7	121,321 (40.2)	84,956 (40.3)	36,365 (40.2)	
	>= 8	42,636 (14.1)	29,770 (14.1)	12,866 (14.2)	
Surgery	No	168,675 (56.0)	117,958 (55.9)	50,717 (56.1)	0.358
	Yes	132,766 (44.0)	93,052 (44.1)	39,714 (43.9)	
Radiation	Yes	116,199 (38.5)	81,306 (38.5)	34,893 (38.6)	0.786
	No	185,242 (61.5)	129,704 (61.5)	55,538 (61.4)	
Age		65.00 [59.00, 70.00]	65.00 [59.00, 70.00]	65.00 [59.00, 70.00]	0.329
PSA		6.20 [4.70, 9.50]	6.20 [4.70, 9.50]	6.20 [4.70, 9.50]	0.942
Follow-up time		5.92 [3.42, 8.83]	5.92 [3.33, 8.83]	5.92 [3.42, 8.83]	0.993
CSS	Alive or dead of other cause	292,466 (97.0)	204,727 (97.0)	87,739 (97.0)	1
	Dead	8975 ( 3.0)	6283 ( 3.0)	2692 ( 3.0)	

The median follow-up time was estimated using the reverse Kaplan–Meier method.

### Conditional cancer-specific survival analysis

**Fig. 3:**
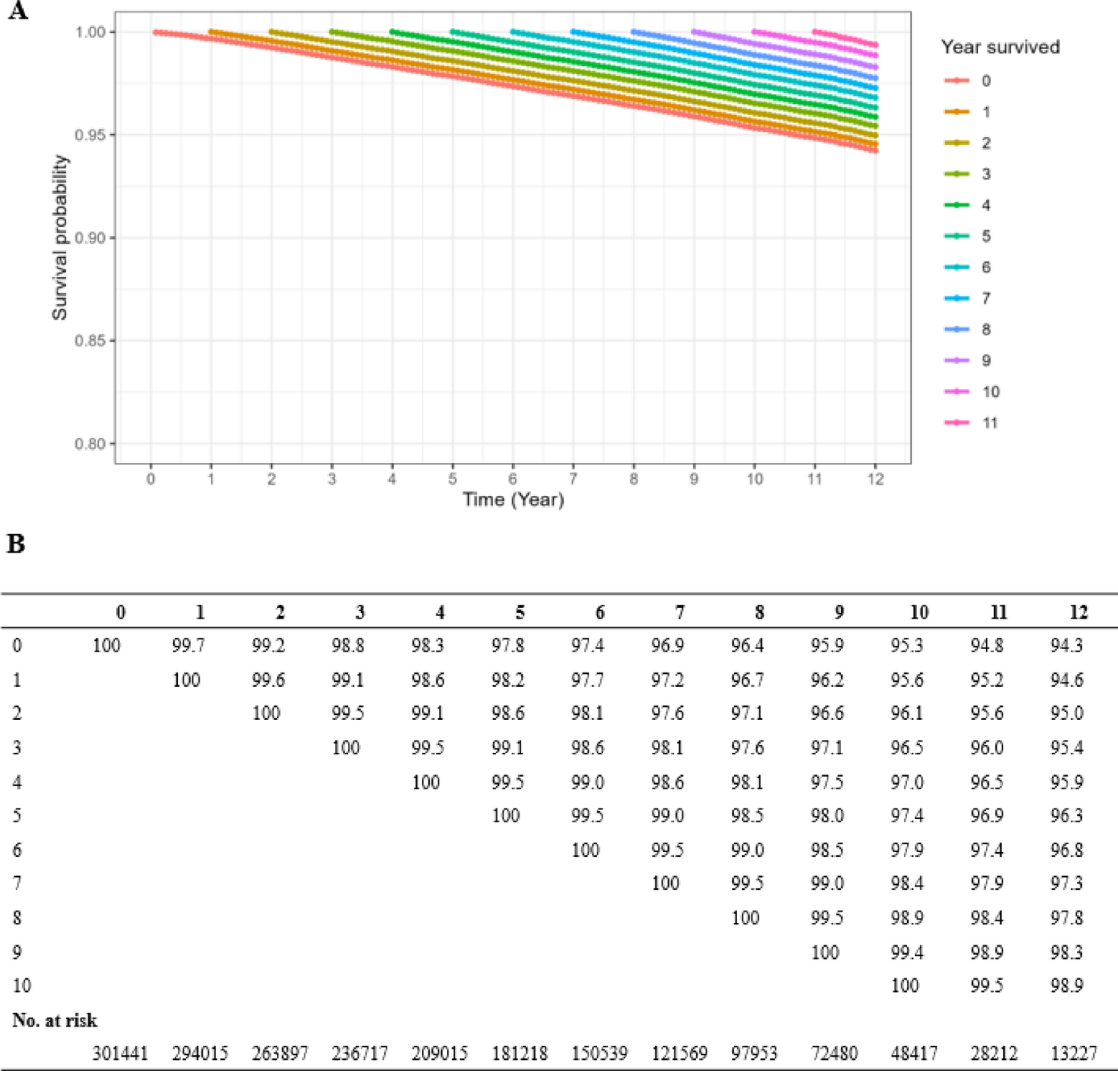
Conditional cancer-specific survival (C-CSS) estimated using Aalen Johansen estimator at 12 years after surviving 0 ~ 10 years in prostate cancer patients. Conditional survival curves (**A**) and their updated survival data adjusted for survived time (**B**).

As of the data cutoff date, 8,975 (3.0%) patients in this study had died from prostate cancer. Survival curves showed that patients had a CSS of 97.8% (95% confidence interval (CI): 97.8-97.9%), 95.3% (95% CI 95.2-95.4%), and 94.3% (95% CI 94.1–94.4) at 5, 10 and 12 years, respectively. The other-cause-related mortality rates of patients at 5, 10, and 12 years were 5.4% (95% CI 5.3–5.5%), 14.4% (95% CI 14.2–14.6%), and 18.9% (95% CI 18.6–19.1%), respectively. The analysis of CS was used to evaluate C-CSS in prostate cancer patients who survived long-term (see Fig. 3). Figure 3A shows how each bend represents the variation in the patient’s CSS with every extra year of survival. Furthermore, the chart documented CSS at every subsequent time interval, with each line indicating CSS post-patients who lived x years and each vertical line indicating CSS at various follow-up times (Fig. 3B). Analysis of C-CSS indicated that the survival of patients would increase steadily with each successive year of survival. Over the course of 12 years, the cumulative survival rate of patients steadily rose from the starting point of 94.3–99.4% with each passing year of follow-up (Fig. 3A).

### Development and validation of the CS-nomogram

**Fig. 4:**
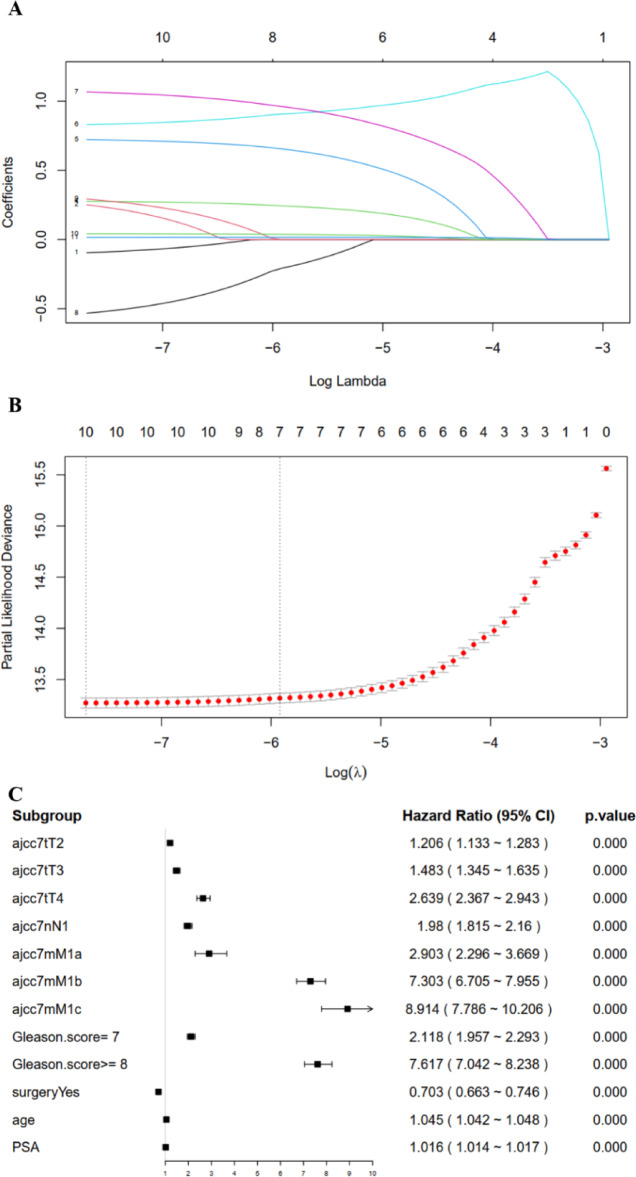
Predictor screening. (**A**) and (**B**). The least absolute shrinkage and selection operator (LASSO) regression and 10-fold cross validation for screening predictors. (**C**). Multivariable Cox regression forest plot showing the effect of predictors on cancer-specific survival (CSS) of prostate cancer. The *p*-value was derived using the Wald test. No significant violations of the proportional hazards assumption were detected (all* p*-values > 0.05, global *p* = 0.120). HR, hazard ratio; CI, confidence interval.

**Fig. 5:**
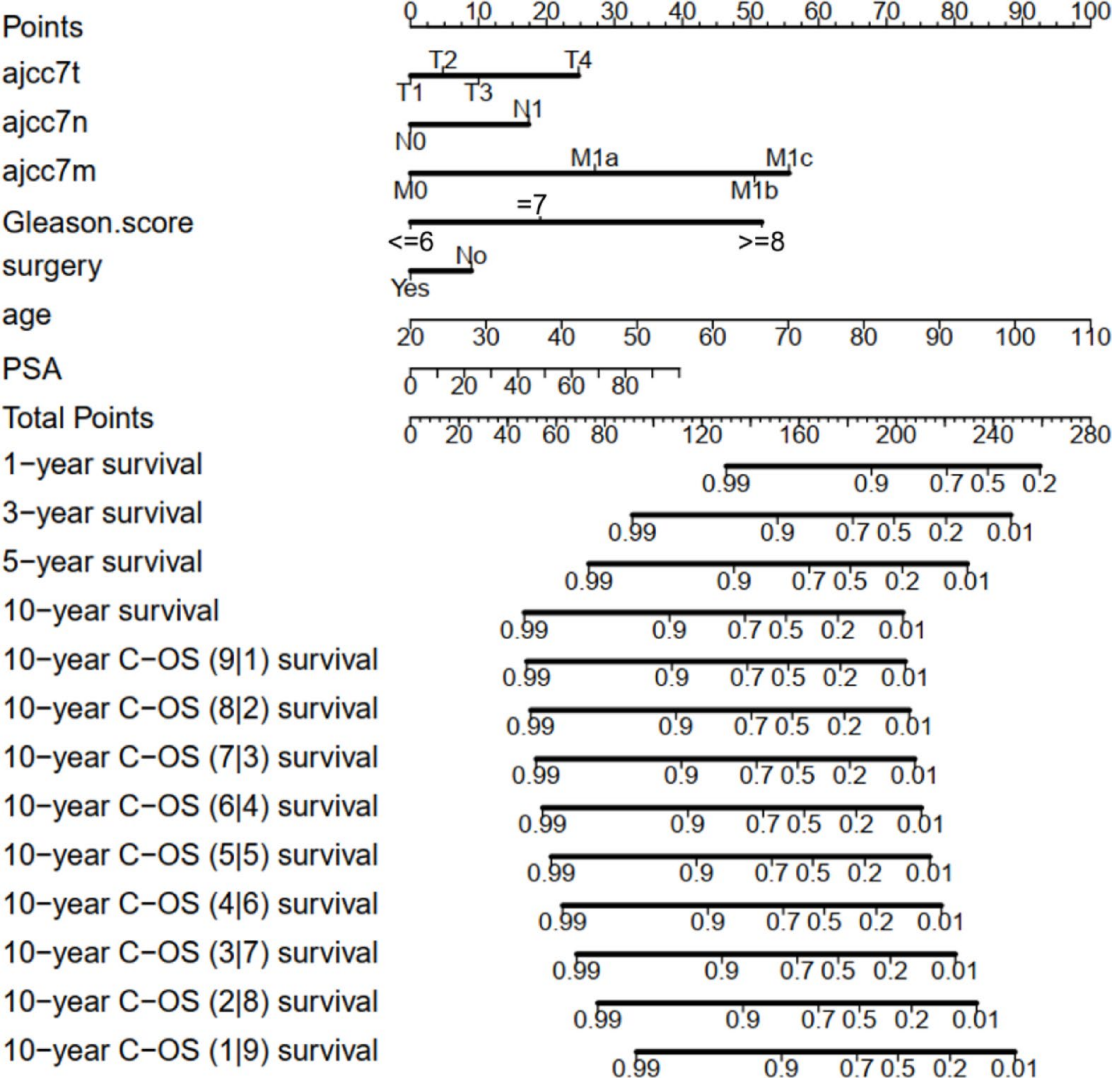
Conditional survival nomogram (CS-nomogram) for predicting 1-, 3-, 5-, and 10-year cancer-specific survival (CSS) and 10-year conditional CSS (C-CSS) for prostate cancer.

Seven clinicopathological factors were identified as predictors by LASSO regression: T stage, N stage, M stage, Gleason score, surgery, PSA level and age (Fig. 4A, B). The forest plot from the multivariable Cox regression analysis indicated that the predictors had a significant impact on the CSS of prostate cancer, leading to the development of the CS-nomogram (*P* < 0.0001, Fig. 4C). Unlike traditional prediction models, the CS-nomogram in this study took into account conditional survival, and patients were able to obtain not only 1-, 3-, 5- and 10-year CSS after inputting individualized clinicopathological factors but 10-year C-CSS based on the number of years they have survived since diagnosis (Fig. 5).

**Fig. 6:**
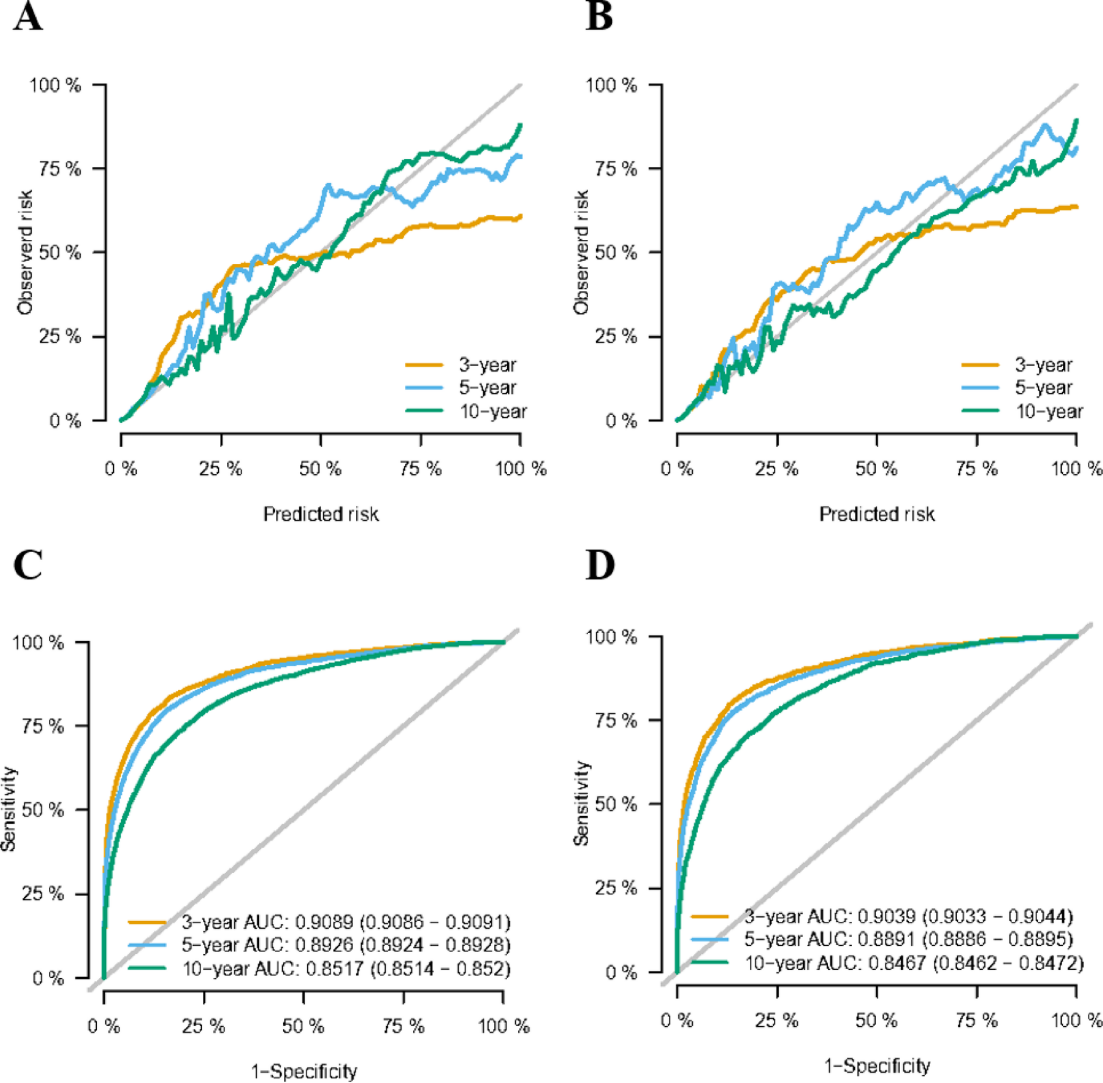
Model evaluation and validation. Calibration plots (**A**) and (**B**), Time-dependent receiver operating characteristic (ROC) curves (**C**) and (**D**) for assessing the accuracy and discrimination of the conditional survival nomogram (CS-nomogram) at 3-, 5- and 10-years, respectively.

**Fig. 7:**
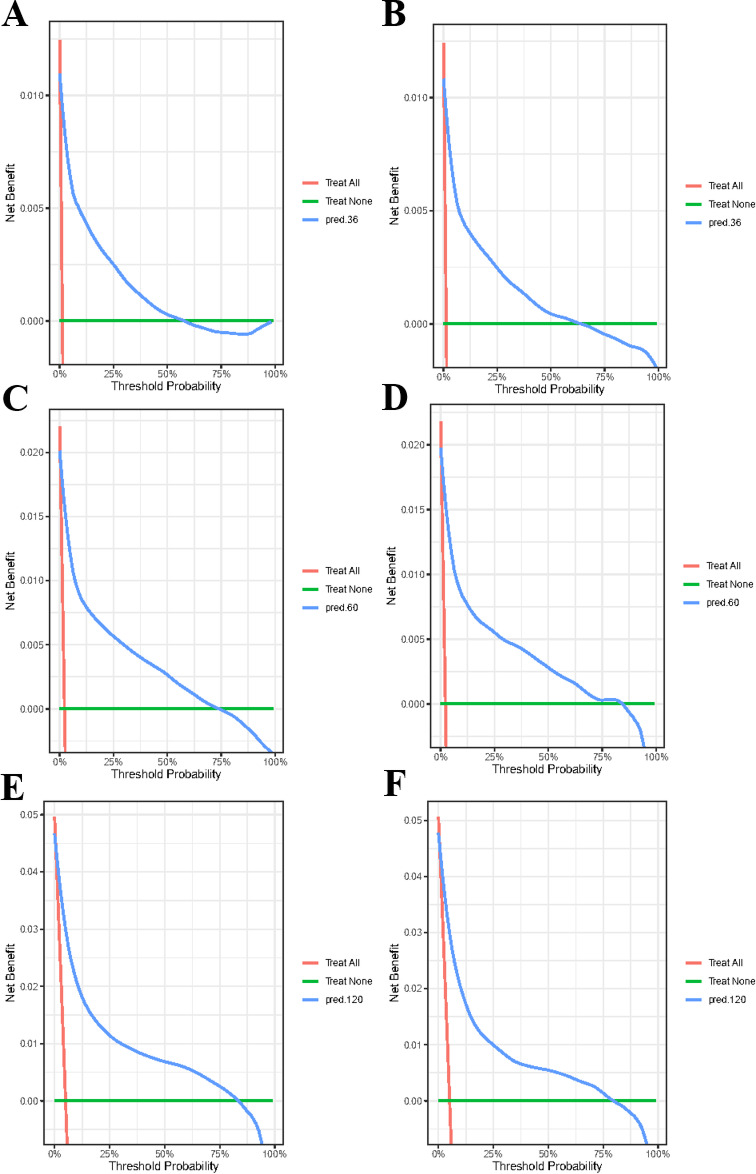
Decision curve analysis (DCA) curves on the training data (**A**. 3-year, **C**. 5-year, **E**. 10-year) and validation data (**B**. 3-year, **D**. 5-year, **F**. 10-year).

This research evaluated how well the model performed in terms of differentiation, precision, consistency over time, and practicality. The C-index values obtained in the training and validation sets were 0.871 (95% CI 0.868–0.874) and 0.869 (95% CI 0.866–0.872), respectively. The calibration plots for these sets at 5, 10, and 12 years demonstrated strong concordance between the CS-nomogram predictions and actual outcomes (Fig. 6A, B). Additionally, the time-dependent ROC curves and C-indexes over 12 years confirmed the model’s robustness in predicting long-term survival (Fig. 6C, D and Supplementary Fig. 1). Furthermore, the DCA plots indicated a consistent positive net gain with the implementation of medical treatments based on the CS-nomogram (Fig. 7).

## Discussion

The results of CS analysis showed that the CSS of prostate cancer patients improved gradually with the years growing. To construct personalized and real-time survival evaluation, we developed a CS-nomogram for those long-term survivors. The results indicated that our model exhibited excellent predictive performance and may provide valuable references to support clinical decision making for prognosis evaluation, follow-up planning, and treatment strategy selection.

Despite being one of the most prevalent cancers among men worldwide, prostate cancer has seen a decline in mortality rates due to advancements in PSA screening and treatment methods^3,4^. The trends in the proportion of advanced T, N, and M stages over time were consistent with those reported in previous literature^20^. Nevertheless, the understanding of conventional survival calculation continues to be difficult. Traditional estimation focuses solely on the initial diagnosis time as a reference point, neglecting to consider the actual duration of the patient’s survival^21^. Thus, the prognosis remains consistent whether it is determined at the initial diagnosis or after the patient has lived for multiple years. The alteration in survival rates over time has attracted considerable attention^22^. Analysis of CS has the potential to provide patients with increased optimism. At the time of the first diagnosis, a patient’s chance of surviving for 12 years was 94.3%. Following his commitment to treatment and regular checkups, he successfully reached the 5-year mark, at which point the CS analysis indicated a 2.1% increase in his 10-year survival rate. Prior studies have shown that if conditional relative survival rates surpass 95%, patient survival and the risk of other cause death are comparable to that of the general population within the same age range^23–25^. Among prostate cancer patients who survived for an extended period in this research, those who lived beyond 5 years had a less than 5% chance of dying from prostate cancer within 7 years. The positive results could alleviate patients’ worries, boost their belief in battling cancer, and enhance their overall well-being. Furthermore, becoming proficient in this ever-changing survival model could assist in developing efficient monitoring techniques for prostate cancer in relation to the length and strength of post-treatment observation.

Another advantage of our research was the incorporation of personalized real-time forecasting of survival. CS predicted survival results in real-time for patients but did not consider personalization. A notable contrast was observed in the outlook for prostate cancer between early and late stages. In order to rectify this inadequacy, we employed the CS-nomogram, which considered the personalized clinicopathological factors of the individual. In this research, T stage, N stage, M stage, Gleason score, surgery, PSA level and age were utilized as predictors, which have been proven to be closely linked to prognosis and play a significant role in determining treatment approach^26,27^. The presence of lymph nodes and distant metastases indicated an advanced stage of the disease, leading to a grim prognosis. After verifying the presence of these predictors, we created a nomogram and included CS in order to offer the initial personalized real-time prognostic instrument for individuals with prostate cancer. The benefit of using the CS-nomogram was the personalized adjustment of survival prediction according to the duration of time that survivors have lived since being diagnosed. Earlier nomograms for prostate cancer only provided estimates for a fixed survival rate and did not account for variations in survival rates over time following long-term patient survival^28^. The dynamic evaluation method provides a promising tool for subsequent disease progression monitoring.

The findings of the ProtecT^29^ trial suggest that treatment has a limited impact on the improvement of the prognosis of patients with localized prostate cancer. However, our study observed that patients who received surgery tended to have better outcomes. It is noteworthy that this study population includes both localized and metastatic prostate cancer patients. The differences in the impact of treatment on prognosis between the two studies may be attributed to variations in the characteristics of the patient populations. For patients with metastatic prostate cancer, such treatments may offer greater benefits by reducing tumor burden and more effectively controlling disease progression.

Despite the strong predictive performance of our model, several limitations should be acknowledged. Advances in imaging techniques, such as MRI and PET scans, may shift the distribution of TNM staging by detecting more early-stage cases. Furthermore, the enhanced precision afforded by these techniques in evaluating tumor extent, lymph node involvement, and distant metastases may lead to refined TNM staging. As a result, differences between historical data and current TNM staging or ISUP classification may affect the generalizability of models. As advances in risk profiling and prostate MRI have led to more selective and personalized approaches to biopsy, the patient population in this study may have different clinical characteristics compared to those identified in contemporary practice. These factors may introduce systematic bias into the model’s predictions, potentially leading to risk overestimation in early-stage patient prognoses. To ensure the model’s predictive accuracy, recalibration—including updating input variables and retraining the model—is necessary before clinical deployment^30^. The patient selection process may lead to potential bias, which can impact the application of the model. Besides, although our nomogram was internally validated, external validation was not conducted due to the lack of independent datasets. Therefore, future studies involving external validation in at least two independent cohorts are warranted to confirm its robustness and applicability. Another limitation is that the CS of patients depends heavily on the disease characteristics at baseline, treatment choice, and follow-up regimen. It seems likely that the survival times represent a selection of localized patients over time, which may prohibit the clinical use of the nomogram. Future work incorporating stratified modeling by disease stage and treatment category, as well as prospective validation in more homogeneous patient subgroups, will be critical to improving the clinical utility and precision of the tool.

In this study, the CS nomogram has been validated internally, which shows its stability and accuracy in predicting patient CSS in real-time. The calibration curves that almost coincided with the ideal curve, the ROC curves that remained stable within 3-, 5-, and 10 years and the DCA that showed high net benefits demonstrated its power. Thus, this novel model provided prognostic information consistent with real-time follow-up and may offer potential clinical utility, pending further prospective validation.

## Electronic supplementary material

Below is the link to the electronic supplementary material.

Supplementary Material 1

## Data Availability

Information on individuals with prostate cancer was obtained from the April 2019 SEER Program update provided by the National Cancer Institute and is accessible to the public.(https://seer.cancer.gov/data/access.html).
