# Supplementary figures and images for: Conditional survival analysis and real-time prognosis prediction for prostate cancer patients
Source: Sci Rep. 2025 May 28;15:18711. doi: 10.1038/s41598-025-00420-9 (PMC12119834; doi:10.1038/s41598-025-00420-9)

## Supplementary Figure 1


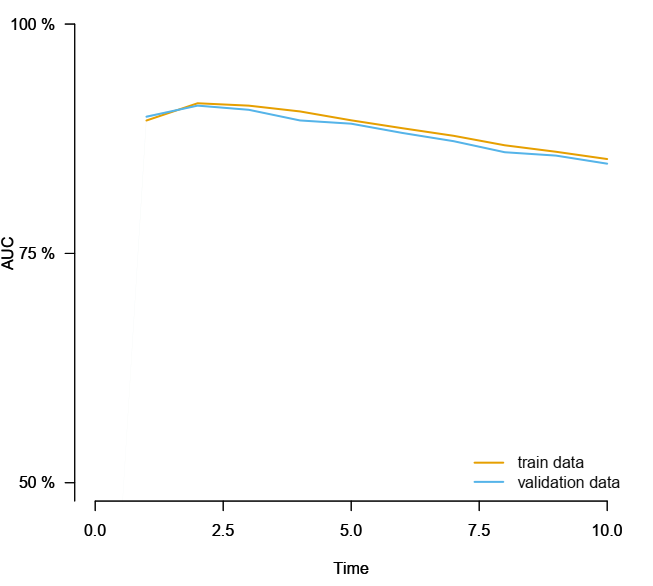


**Supplementary Figure 1** Time-dependent AUC of the CS-nomogram

Supplement: Supplementary file 1 — Supplementary Material 1 [file 41598_2025_420_MOESM1_ESM.docx]
